# Heterogeneity and the determinants of PM_2.5_ in the Yangtze River Economic Belt

**DOI:** 10.1038/s41598-022-08086-3

**Published:** 2022-03-09

**Authors:** Siyou Xia, Xiaojie Liu, Qing Liu, Yannan Zhou, Yu Yang

**Affiliations:** 1 Institute of Geographic Sciences and Natural Resources Research, Chinese Academy of Sciences, Beijing, 100101 China; 2 College of Resources and Environment, University of Chinese Academy of Sciences, Beijing, 100049 China; 3 School of Geographic and Oceanographic Sciences, Nanjing University, Nanjing, 210023 China; 4 School of Geography and Planning, Sun Yat-Sen University, Guangzhou, 510275 China

**Keywords:** Environmental sciences, Environmental social sciences

## Abstract

Haze has reached epidemic levels in many Chinese cities in recent years. Few studies have explored the determinants and heterogeneity of PM_2.5_. This paper investigates the spatiotemporal characteristics of PM_2.5_ through spatial analytical methods based on aerosol optical depth data from the Yangtze River Economic Belt (YREB) between 2000 and 2017. Geographically weighted regression and geodetector models were applied to assess the heterogeneity of key factors influencing PM_2.5_. The results indicate that the annual concentrations of PM_2.5_ in the YREB were 23.49–37.37 μg/m^3^, with an initial increase and a later decrease. PM_2.5_ pollution showed a diagonal high spatial distribution pattern in the northeast and a low spatial distribution in the southwest, as well as a noticeable spatial convergence. The spatial variability of PM_2.5_ was enlarged, and its main fractal dimension was in the northeast-southwest direction. There were clear spatiotemporal variations in the impacts of natural and anthropogenic factors on PM_2.5_. Our findings contribute to a better understanding of the impact mechanisms of PM_2.5_ and the geographic factors that form persistent and highly polluted areas and imply that more specific coping strategies need to be implemented in various areas toward successful particulate pollution prevention and control.

## Introduction

Haze is present in most urban regions of the world and is a global environmental problem. The rapid increase in the urbanization and industrialization of many cities in China has caused haze pollution, which is dominated by the enhanced concentration of PM_2.5_ (particulate matter with an aerodynamic diameter ≤ 2.5 μm). PM_2.5_ has significant adverse impacts on public health, the environment, and climate because of its small diameter, high activity, and ability to transport noxious substances in the air with long residence times^1–3^. Epidemiological studies have confirmed that PM_2.5_ can induce various respiratory and cardiovascular diseases, impair the body’s immune system, and increase the mortality of people exposed to it^4,5^. More than 1.3 million people in China die prematurely every year due to prolonged exposure to polluted air, which is approximately 40% of the global total^6^. The International Agency for Research on Cancer (IARC) classified PM_2.5_ as a human carcinogen in 2013^7^. Moreover, continuous haze damages the overall image of a city and weakens the attraction of tourism, talent, and investments, which restricts the sustainable development of the economy^8–10^. The issues related to cleaning the air have aroused attention both domestically and globally.

PM_2.5_ has attracted significant attention from the atmospheric and climate science community, and many studies have explored environmental issues related to it, including spatiotemporal distribution and drivers. Research on the spatial patterns of PM_2.5_ mainly focuses on environmental coping policies by identifying its distribution patterns and spatial effects. These studies reveal that PM_2.5_ has regional, cumulative, and compound effects and possesses significant spatiotemporal variability^11–13^. In addition, PM_2.5_ is not restricted to the local environment and can be diffused or transferred to neighboring areas largely through external forces, such as atmospheric circulation causing spillover effects^14^. The identification and estimation of PM_2.5_ with spatial characteristics should be studied by means of spatial analytical methods rather than by traditional statistical theory based on independent observations^15^. Spatial statistics started in the 1970s with the goal of understanding spatial dependence, spatial association, and other relations among data related to geographical locations with wide applications in many fields. Accurate identification of PM_2.5_ determinants can provide a strong theoretical basis for the prevention and control of air pollution. Therefore, many scholars are committed to understanding source analyses, emission inventories, chemical conversion, and regional transport of PM_2.5_ and have achieved significant results. In general, PM_2.5_ is considered to be emitted from local sources, including primary sources, such as traffic fumes, industrial activities, soil dust, biomass, and coal combustion, and secondary sources of gaseous pollutants (such as SO_2_, NOx, and NH_3_) formed by complex chemical reactions^10,16–18^. Thus, a local PM_2.5_ concentration change is the result of the combined action of natural and human factors^19,20^. Natural conditions, such as meteorology, topography, and vegetation, play important roles in the generation, accumulation, transfer, diffusion, and settlement of PM_2.5_ and profoundly affect its local concentration^20,21^. With respect to the influence of socioeconomic factors related to anthropogenic activities on the distribution of PM_2.5_, studies suggest that energy-intensive economic growth and nonecological urbanization increase PM_2.5_ concentrations, implying that economic development, urbanization, industrialization, land use, and energy use mixtures and efficiency can affect urban air quality^13,22–24^. These studies provide many insights into urban particle pollution from the perspectives of artificial and natural conditions.

The Yangtze River Economic Belt (YREB) is one of China’s most crucial economic and ecological corridors that connects three national urban agglomerations of the Sichuan–Chongqing, the middle reaches of the Yangtze River, and the Yangtze River Delta (YRD). The YREB accounts for more than 40% of China’s population and GDP, and its unique geographical advantages and vast economic hinterland make it the region with the greatest economic growth potential in the next 30 years^25^. However, urban air quality along the Yangtze has been deteriorating and has suffered from varying degrees of haze pollution due to continuous and intense industrial and human activities^26^. Recently, some areas in the middle-lower reaches of the Yangtze River have had more than 100 haze d year^−1^, with some cities even exceeding 200 d year^−1^^27^. In the upper reaches of the Yangtze River, the ecological environment is relatively vulnerable as economic development is scaled up^28^. The alleviation of haze pollution involves the integrity of the ecological and environmental system and the quality of local people’s lives^9^. In 2014, the development of the YREB was given national strategy status, leading to the fact that potential conflicts between economic growth and environmental conservation may be more prominent than ever before^29^. In 2018, President Xi emphasized the protection of the eco-environmental status of the YREB. Hence, the eco-environmental conservation of the Yangtze River is a priority for the government of China^25^.

Until now, studies on the environmental problems of the YREB have mainly focused on the macro-level, including environmental quality and risk assessment, a low-carbon economy, and a sustainable development strategy^9,28^. Studies on environmental problems at the micro-level focus more on water pollution, while studies on air pollution are still insufficient^27^. As the China National Environmental Monitoring Centre (CNEMC) did not include PM_2.5_ in its monitoring index system until 2012, relatively few studies focused on PM_2.5_ in the YREB prior to 2012^30^. The CNEMC has begun widespread real-time monitoring of PM_2.5_ since 2013. Furthermore, the use of remote sensing data for long-term time series research was limited to local areas and specific cities, and studies on factors of influence were mostly in terms of the overall perspective^31^. Obvious spatial differences in the natural conditions and socioeconomic development of various regions exist, and hence, a global analysis cannot reveal the spatial heterogeneity of the effects of various factors^32^. On the other hand, the majority of research has not conducted spatial analysis from a geographical viewpoint. Although some research has considered the spatial dependence of PM_2.5_ pollution, it has still neglected the spatial differentiation of the PM_2.5_ level at the urban agglomeration scale and lacked analysis of the interactive mechanism between factors, which may have led to incomplete and biased conclusions^33^.

In this study, we attempt to fill the aforementioned knowledge gaps. We consider 130 cities within the YREB as the research area and employ geostatistical and spatial analytical methods to summarize the spatiotemporal evolutionary features of PM_2.5_. Furthermore, we adopt a geographically weighted regression model and geographical detectors to quantitatively analyze the spatial differentiation and interactive effects of socioeconomic and natural conditions on PM_2.5_ during various periods. Thus, our study is significant in terms of meeting the demand for air quality improvements in the YREB and sheds light on implementing effective urban PM_2.5_ pollution abatement policies.

## Data and methods

### Study area

**Figure 1:**
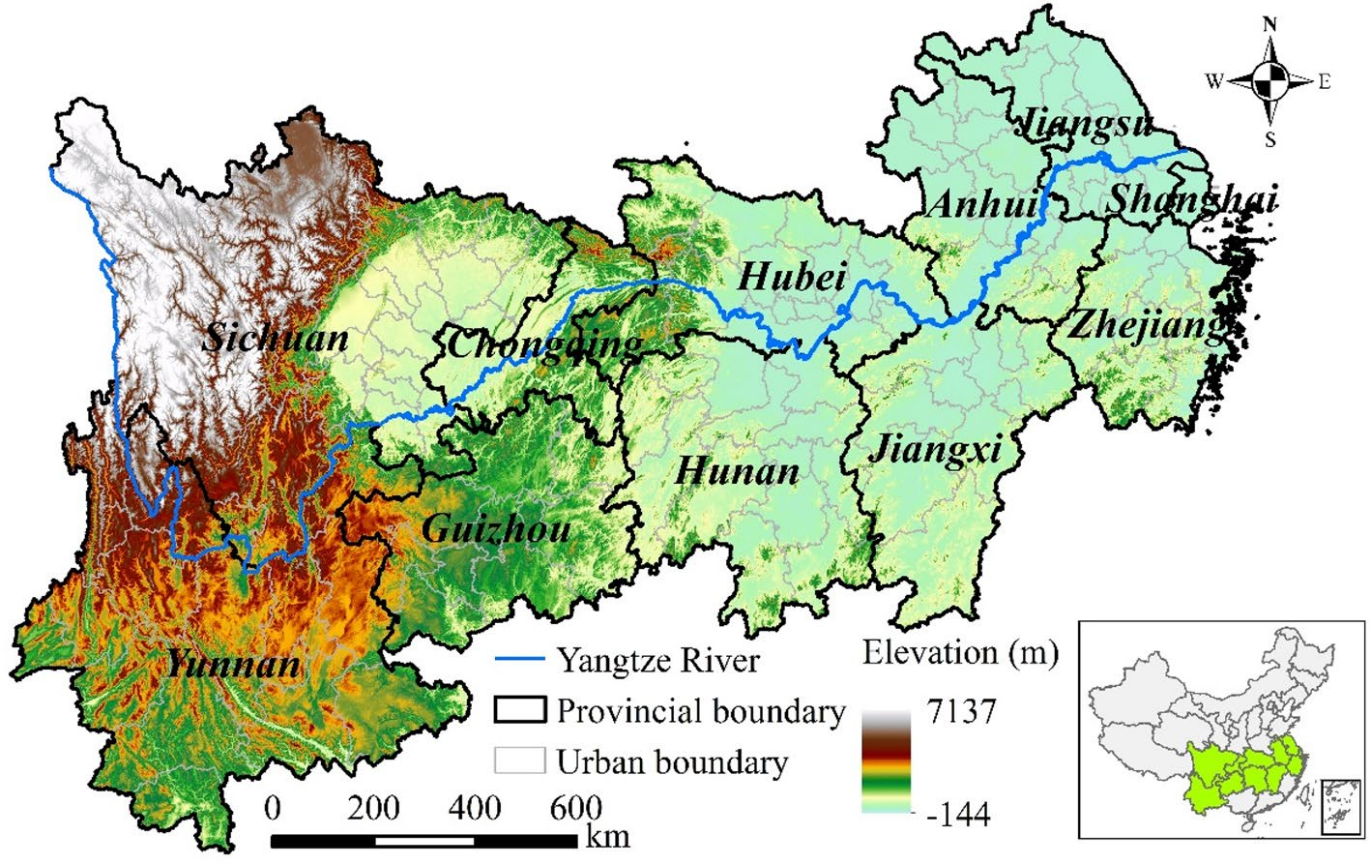
General overview of the YREB. Standard map services are provided by the Ministry of Natural Resources of China (http://bzdt.ch.mnr.gov.cn/), GS (2020)4619.

The YREB spans the eastern, central, and western regions of China, including nine provinces (Zhejiang, Jiangsu, Jiangxi, Anhui, Hubei, Hunan, Sichuan, Yunnan, and Guizhou) and two municipalities (Shanghai and Chongqing) (Fig. 1). The YREB comprises an area of 2.05 × 10^6^ km^2^, accounting for 21.3% of the country’s land area. Moreover, its economy and population density are 6.2 and 4.5 times the national average, respectively, establishing its incomparable role in China’s development strategies^8^. However, the YREB is an ecologically vulnerable zone, and hence, assessing PM_2.5_ pollution in the region is of great strategic significance for national ecological security and regional sustainable development^28^.

### Data acquisition and processing

Our data sources have four components: (1) PM_2.5_ concentration data from satellite retrievals. The annual concentrations of PM_2.5_ were obtained from the global surface raster with a resolution of 0.01° × 0.01°, which was released by the Atmospheric Composition Analysis Group (ACAG) (https://sites.wustl.edu/acag/). This PM_2.5_ remote sensing inversion dataset has the largest global coverage, the longest time span, and the highest accuracy, and it has been widely verified and effectively applied in China^34^. To verify the accuracy of the dataset in the YREB, we calculated the annual concentration of each station through real-time monitoring data of the YREB from 2015 to 2017 obtained from the CNEMC (http://www.cnemc.cn) and correlated it with the PM_2.5_ concentration from the remote sensing inversion data at the corresponding position. The correlation coefficient was 0.82 and significant, indicating a strong correlation and consistency between these datasets. (2) Data on natural parameters include wind, precipitation, vegetation, and topography^21,35^. The normalized differential vegetation index (NDVI) is the best indicator of vegetation coverage and growth status, and the original data were derived from the National Aeronautics and Space Administration (NASA) (https://www.nasa.gov/), while other supporting data were obtained from the Resource and Environment Data Cloud Platform (http://www.resdc.cn). (3) Socioeconomic data includes per capita GDP, population density, economic density, urbanization rate, industrial structure, and energy consumption^33,36^. Studies have shown that there is a significant linear correlation between nighttime light data from the Defense Meteorological Satellite Program/Operational Line Scanner (DMSP/OLS) and energy consumption^37^. We converted the light intensity to gray pixel values and used the sum of all DMSP/OLS raster gray values in each region as an indicator of energy consumption in the region. The DMSP/OLS dataset was obtained from the National Oceanic and Atmospheric Administration (NOAA) (https://www.ngdc.noaa.gov), and the remaining data were collected from the China Urban Statistics Yearbook published by the National Bureau of Statistics (http://www.stats.gov.cn/). (4) Geographic information data includes the spatial vector map, which was derived from the National Catalog Service for Geographic Information (http://www.webmap.cn).

As multicollinearity in the selected variables may cause information redundancy, we adopted the variance inflation factor (VIF) for multicollinearity diagnosis with a threshold of 5. We excluded the indicators of economic density and energy consumption because their VIFs were greater than 5. Thus, 8 explanatory variables were used in the model, including per capita GDP (*pgdp*), population density (*popd*), urbanization rate (*urba*), secondary industry share (*indu*), annual wind speed (*wind*), annual precipitation (*prec*), NDVI (*ndvi*), and topographic relief (*topo*). To reduce data heteroscedasticity, all the indicators were treated with Z–standardization.

## Methods

### Spatial autocorrelation analysis

Tobler’s first law of geography states that everything is related to everything else, but nearby things are more related than distant things^15^. Therefore, we adopted the classical spatial autocorrelation method to quantitatively measure the spatial dependence of PM_2.5_ in neighboring regions. Global Moran’s *I* is written as^38^:

1 $$I\frac{{n\sum\limits_{i = 1}^{n} {\sum\limits_{j = 1}^{n} {W_{ij} \left( {x_{i} - \overline{x}} \right)\left( {x_{j} - \overline{x}} \right)} } }}{{\sum\limits_{i = 1}^{n} {\sum\limits_{j = 1}^{n} {W_{ij} \sum\limits_{i = 1}^{n} {\left( {x_{i} - \overline{x}} \right)^{2} } } } }}$$

where *x*_*i*_ and *x*_*j*_ are the observations of spatial units *i* and *j*, respectively; $\overline{x }$ is the mean of *n* locations; and *W*_*ij*_ is a spatial weight matrix. *I* ∈ [− 1, 1], where *I* > 0 indicates positive correlation, *I* < 0 indicates negative correlation, and *I* = 0 indicates mutual independence.

### Spatial variogram analysis

The spatial variogram is a geostatistical method to describe the structure and randomness of regionalized variables and is often used to effectively measure the spatial structure characteristics and variation pattern of geographical variables^39^. The variogram is expressed as:

2 $$\gamma \left( \lambda \right) = \frac{1}{2N\left( \lambda \right)}\sum\limits_{i = 1}^{N\left( \lambda \right)} {\left[ {\xi \left( {x_{i} } \right) - \xi \left( {x_{i} + \lambda } \right)} \right]^{2} }$$

where *ξ*(*x*_*i*_) and *ξ*(*x*_*i*_ + *λ*) are the values of the regionalized variables at points *x*_*i*_ and *x*_*i*_ + *λ*, respectively, and *N*(*λ*) is the sample size with separation distance *λ.*

There are three major parameters derived from the variogram model^39–41^. The nugget parameter (*C*_0_) is a random spatial variance; the partial sill parameter (*C*) is a structural spatial variance; the sill parameter (*C*_0_ + *C*) represents the total degree of spatial variation. The nugget effect (*C*_0_/(*C*_0_ + *C*)) indicates whether regional or local factors are more important for PM_2.5_. If the nugget effect is less than 0.25, it indicates strong spatial correlation; if it is between 0.25 and 0.75, it shows moderate spatial correlation; and if it is greater than 0.75, it indicates weak spatial correlation^41^. The range parameter (*A*_0_) represents the maximum spatial distance of the correlation.

The fractal dimension is another important parameter that characterizes the variogram, and its value is determined by the relationship between the variogram *γ*(*λ*) and distance *λ*:

3 $$2\gamma \left( \lambda \right) = \lambda^{{\left( {4 - 2D} \right)}}$$

where *D* is the slope of the double logarithm linear regression equation; the higher the value is, the higher the heterogeneity caused by the spatial autocorrelation. The closer the value is to 2, the more balanced the spatial distribution.

### Geographically weighted regression modeling (GWR)

Geographically weighted regression (GWR) is a spatial regression model based on the idea of local smoothness^42^. This technique constructs an independent equation for each unit in the study area and incorporates the spatial attributes of data into the regression model so that the relationship between variables can change with the change in spatial location, thus reflecting the spatial nonstationarity of parameters in different regions. Its formula is as follows^16^:

4 $$y_{i} = \varphi_{0} \left( {\omega_{i} ,\alpha_{i} } \right) + \sum\limits_{i = 1}^{p} {\varphi_{p} \left( {\omega_{i} ,\alpha_{i} } \right)x_{ip} + \varepsilon_{i} }$$

where *x*_*ip*_ is a dimensional interpretation variable matrix; (*ω*_*i*_, *α*_*i*_) represents the longitude and latitude coordinates at the *i*th observation point; *φ*_*p*_(*ω*_*i*_, *α*_*i*_) is the regression coefficient of the *i*th observation point; and *ε*_*i*_ is a random error term.

### Geographical detector technique

The geographical detector technique is a set of statistical methods to detect spatially stratified heterogeneity and reveal the driving forces behind it^43^. It can detect the possible causal relationship between variables by verifying the consistency of the spatial distribution of two variables^44^. The explanatory power of the factor is measured by the *q* value, and its expression is as follows:

5 $$q = 1 - \frac{{\sum\limits_{h = 1}^{L} {N_{h} \sigma_{h}^{2} } }}{{N\sigma^{2} }}$$

where *L* refers to the strata of variable *Y* (PM_2.5_) or factor *X*; *N*_*h*_ and ${\sigma }_{h}^{2}$ are the number of units and variance of strata *h*, respectively; *N* and σ^2^ are the total number of units and variance, respectively;$q\, \in \,\left[ {0,{ 1}} \right]$ , and the greater the value is, the stronger the explanatory power of this factor is.

The purpose of interactive detection is to assess whether the factors *X*_1_ and *X*_2_ work together to increase or decrease the explanatory power on *Y* or whether the impact of these factors on *Y* is independent. The evaluation method first calculates the *q* value of the two factors *X*_1_ and *X*_2_ acting on *Y*, namely, *q*(*X*_1_) and *q*(*X*_2_), and then calculates the *q* value for their interaction, namely, *q*(*X*_1_ ∩ *X*_2_); finally, *q*(*X*_1_) and *q*(*X*_2_) are compared with *q*(*X*_1_ ∩ *X*_2_). The discriminant criteria can be divided into 5 categories (Table 1).

**Table 1:** Types of interaction between two covariates.

Diagram	Criterion	Interaction
	*q*(*X*_1_ ∩ *X*_2_) < Min(*q*(*X*_1_), *q*(*X*_2_))	Nonlinear weakening
	Min(*q*(*X*_1_), *q*(*X*_2_)) < *q*(*X*_1_ ∩ *X*_2_) < Max(*q*(*X*_1_), *q*(*X*_2_))	Univariate nonlinear weakening
	*q*(*X*_1_ ∩ *X*_2_) > Max(*q*(*X*_1_), *q*(*X*_2_))	Bivariate enhancement
	*q*(*X*_1_ ∩ *X*_2_) = *q*(*X*_1_) + *q*(*X*_2_)	Independent
	*q*(*X*_1_ ∩ *X*_2_) > *q*(*X*_1_) + *q*(*X*_2_)	Nonlinear enhancement

Min(*q*(*X*_1_), *q*(*X*_2_)) is the minimum value between *q*(*X*_1_) and *q*(*X*_2_);  Max(*q*(*X*_1_), *q*(*X*_2_)) is the maximum value between *q*(*X*_1_) and *q*(*X*_2_); *q*(*X*_1_) + *q*(*X*_2_) is the sum of *q*(*X*_1_) and *q*(*X*_2_); *q*(*X*_1_ ∩ *X*_2_) is the interaction between *q*(*X*_1_) and *q*(*X*_2_).

## Results

### Spatiotemporal evolutionary characteristics of PM_2.5_

**Figure 2:**
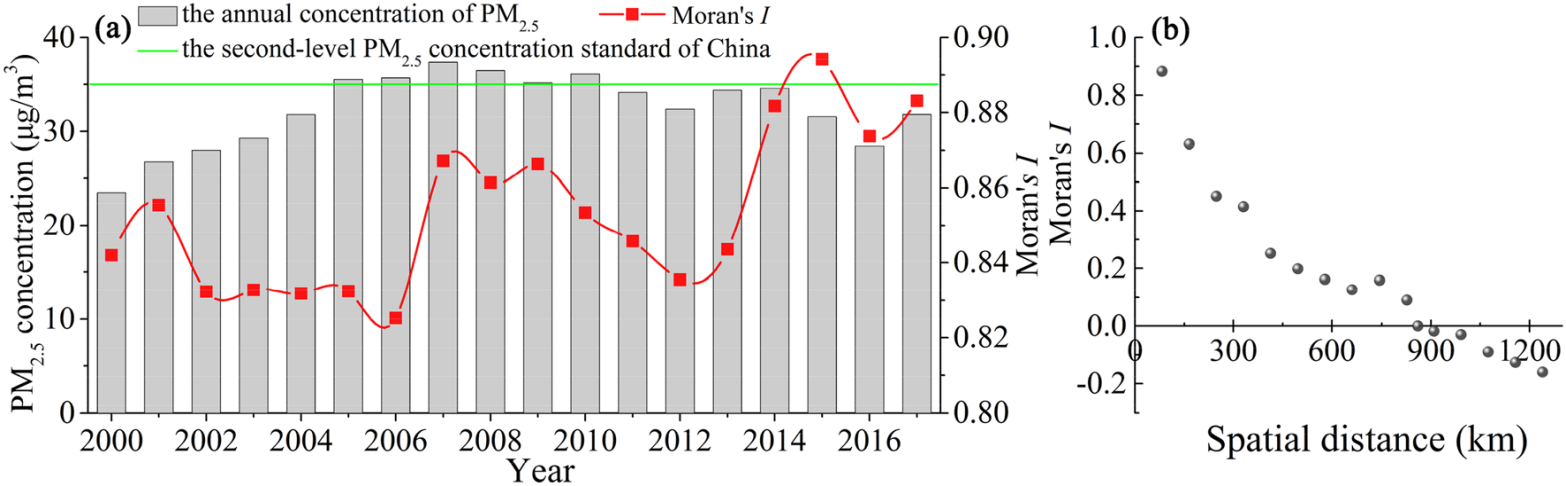
Variation in the PM_2.5_ concentration and Moran’s *I* value in the YREB.

During 2000–2017, the overall PM_2.5_ level in the YREB showed a trend of first increasing and then decreasing (Fig. 2a). In 2005–2010, the annual PM_2.5_ concentration in the YREB was higher than the second-level standard of China of 35 μg/m^3^. During 2000–2007, the PM_2.5_ level increased from 23.49 to 37.37 μg/m^3^, an increase of 59.07%. Afterward, it decreased to 31.79 μg/m^3^ in 2017, a decrease of 14.93%. This improvement may be due to the effects of the national tenth 5-year plan on controlling the total emissions of major pollutants, adjusting the industrial structure, and establishing a monitoring, statistics, and assessment system for energy conservation and pollution emission reduction. It was further reinforced by the implementation of the Action Plan for Air Pollution Prevention and Control in 2013 (http://www.mee.gov.cn).

Figure 2a shows that the sliding interval of the global Moran’s *I* value over the years was [0.825, 0.894], and all the values were significant at the 99% level. This indicates that the PM_2.5_ distribution was not random but was a significant spatial agglomeration. We pursued the association of the evolution rules of spatial correlation with distance and found that the distance threshold to maintain spatial correlation was approximately 870 km (Fig. 2b). Within this spatial range, PM_2.5_ had significant positive interaction effects, which increased with the shortening of distance.

**Figure 3:**
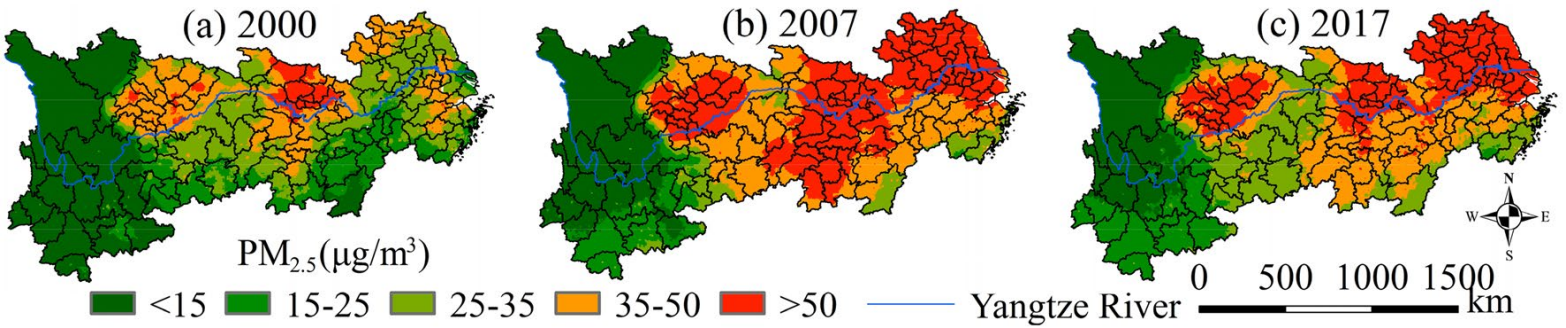
Spatial patterns of PM_2.5_ in the YREB between 2000 and 2017.

Figure 3 displays the spatial patterns and evolution of PM_2.5_ in the YREB from 2000 to 2017. Its main features are as follows: (1) cities with an annual PM_2.5_ level of less than 15 μg/m^3^ were mainly concentrated in ethnic minority areas, where the environmental conditions were relatively good. However, air quality continuously deteriorated, albeit at low levels, which needs attention. (2) The PM_2.5_ level was higher in the lower reaches than in the upper reaches and higher on the north bank than on the south bank, presenting a diagonal spatial distribution pattern with an obvious lowland plain directivity. (3) Urban economic activities and population density were closely related to PM_2.5_ in three centers, namely, the Cheng-Yu area, the Wuhan metropolitan area, and the northern Anhui–Jiangsu region.

### Spatiotemporal variation characteristics of PM_2.5_

The value of the variogram increased with increasing separation distance, indicating that the spatial autocorrelation of PM_2.5_ changed from strong to weak with increasing distance (Table 2). During 2000–2017, the variation range was 625–738 km, and it showed an overall upward trend, implying that the spatial correlation of PM_2.5_ was partly expanded in scope. In addition, the nugget effect indicated that regional-scale factors are more important for the distribution of PM_2.5_.

**Table 2:** Fitting parameters of the PM_2.5_ concentration variogram.

Year	*A*_0_ (km)	*C*_0_	*C*_0_ + *C*	*C*_0_/(*C*_0_ + *C*)	*R*^2^	Residual sum of squares	Optimal fitting model
2000	625.27	0.0029	0.0543	0.0534	0.960	2.061E−04	Gaussian
2007	737.85	0.0036	0.0492	0.0732	0.963	1.444E−04	Gaussian
2017	635.66	0.0011	0.0484	0.0227	0.987	5.271E−05	Gaussian

**Figure 4:**
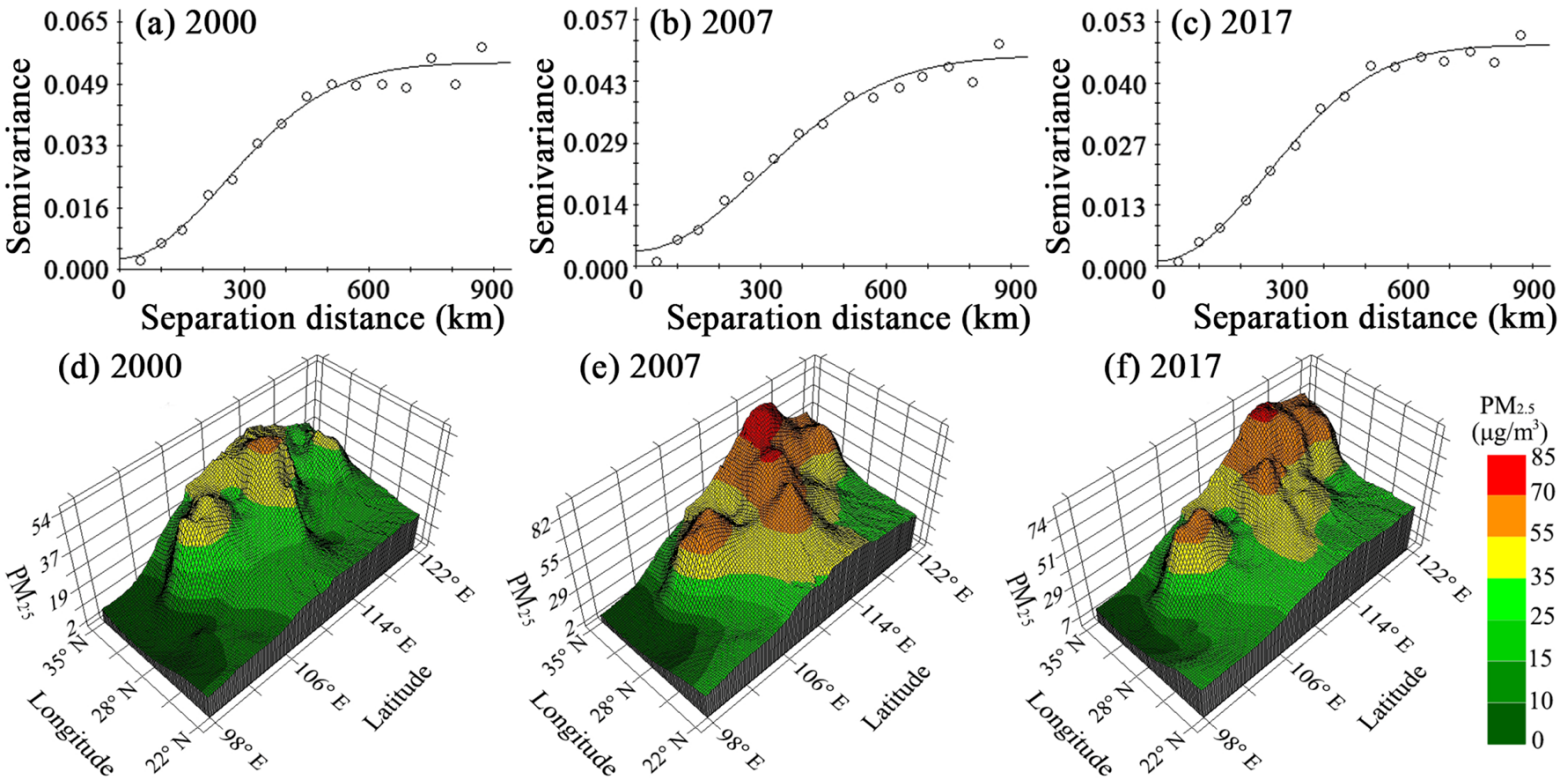
Evolution of PM_2.5_ concentrations in the YREB based on variograms.

In terms of the fractal dimension (Table 3), the isotropic dimension continuously decreased from 1.536 in 2000 to 1.453 in 2017, indicating that the spatial difference in PM_2.5_ was continuously expanding. The northeast-southwest direction had the greatest goodness of fit and the smallest fractal dimension and showed a downward trend. This result denoted that the spatial variation in PM_2.5_ in this direction was continuously strengthened, making it the main direction in terms of spatial difference. The southeast-northwest fractal dimension was the largest, and its decisive coefficient continued to decrease, showing that the spatial difference in PM_2.5_ in this direction continued to weaken and remained relatively evenly balanced. We conducted 3D-kriging interpolation, which further depicted the spatial distribution and evolution morphology of PM_2.5_ (Fig. 4). It was evident that the spatial pattern steadily transitioned from a gradient differentiation to a relatively balanced structure that formed a trend indicating that the middle-lower reaches of the Yangtze River drove the whole basin PM_2.5_ level to increase.

**Table 3:** Variable difference dimension of PM_2.5_ concentrations.

Year	Isotropic		South–North (0°)		Northeast–Southwest (45°)		East–West (90°)		Southeast–Northwest (135°)	
	*D*	*R*^2^	*D*	*R*^2^	*D*	*R*^2^	*D*	*R*^2^	*D*	*R*^2^
2000	1.536	0.959	1.517	0.847	1.430	0.985	1.603	0.918	1.527	0.922
2007	1.482	0.977	1.554	0.763	1.409	0.997	1.464	0.914	1.520	0.756
2017	1.453	0.936	1.509	0.774	1.292	0.985	1.258	0.787	1.567	0.611

## Analysis models of the factors of influence

### GWR model results

The GWR model fitting results are shown in Table 4, in which the adjusted *R*^2^ values are above 0.9, indicating good fitting performance.

**Table 4:** Fitting results of the GWR model.

Variables and parameters	2000	2007	2017
*pgdp*	− 0.2792 to 0.4534*	− 0.1764 to 0.6815**	− 0.1982 to 0.4392*
*popd*	− 0.2327 to 1.2928**	0.0070–0.9061***	− 0.0448 to 1.4807***
*urba*	− 0.2099 to 0.5177**	− 0.0996 to 0.3314*	− 0.1814 to 0.1884**
*indu*	− 0.1569 to 0.1629*	− 0.1711 to 0.2928*	− 0.0612 to 0.1158***
*wind*	− 1.5755 to 0.2788***	− 0.6810 to 0.1470***	− 0.4908 to 0.3147*
*prec*	− 0.8223 to 0.4898***	− 0.3110 to 0.0518*	− 0.2301 to 0.3349**
*ndvi*	− 0.6251 to 0.0474***	− 0.7815 to 0.0027***	− 0.5942 to − 0.0026**
*topo*	− 0.6420 to 0.1005***	− 0.7488 to − 0.1018***	− 1.1785 to − 0.1100***
Bandwidth	3.321	3.627	3.289
AICc	118.931	51.926	16.542
*R*^2^	0.949	0.966	0.977
Adjusted *R*^2^	0.913	0.944	0.960

*, ** and *** indicate significance at the 10%, 5% and 1% levels, respectively.

The regression coefficients of socioeconomic factors, such as per capita GDP, population density, urbanization rate, and industrial structure, were mainly positive. Among them, population density had the largest impact on PM_2.5_ and the most obvious spatial difference, followed by per capita GDP, while the coefficients of urbanization and industrial structure were relatively small. The regression coefficients of natural factors, such as wind, precipitation, vegetation, and topography, were distributed in positive and negative intervals, and the instability was striking in different years. Among them, the coefficient of the distribution interval for topographic relief was the longest, indicating that spatial heterogeneity was the largest.

### Spatial heterogeneity of factors of influence

**Figure 5:**
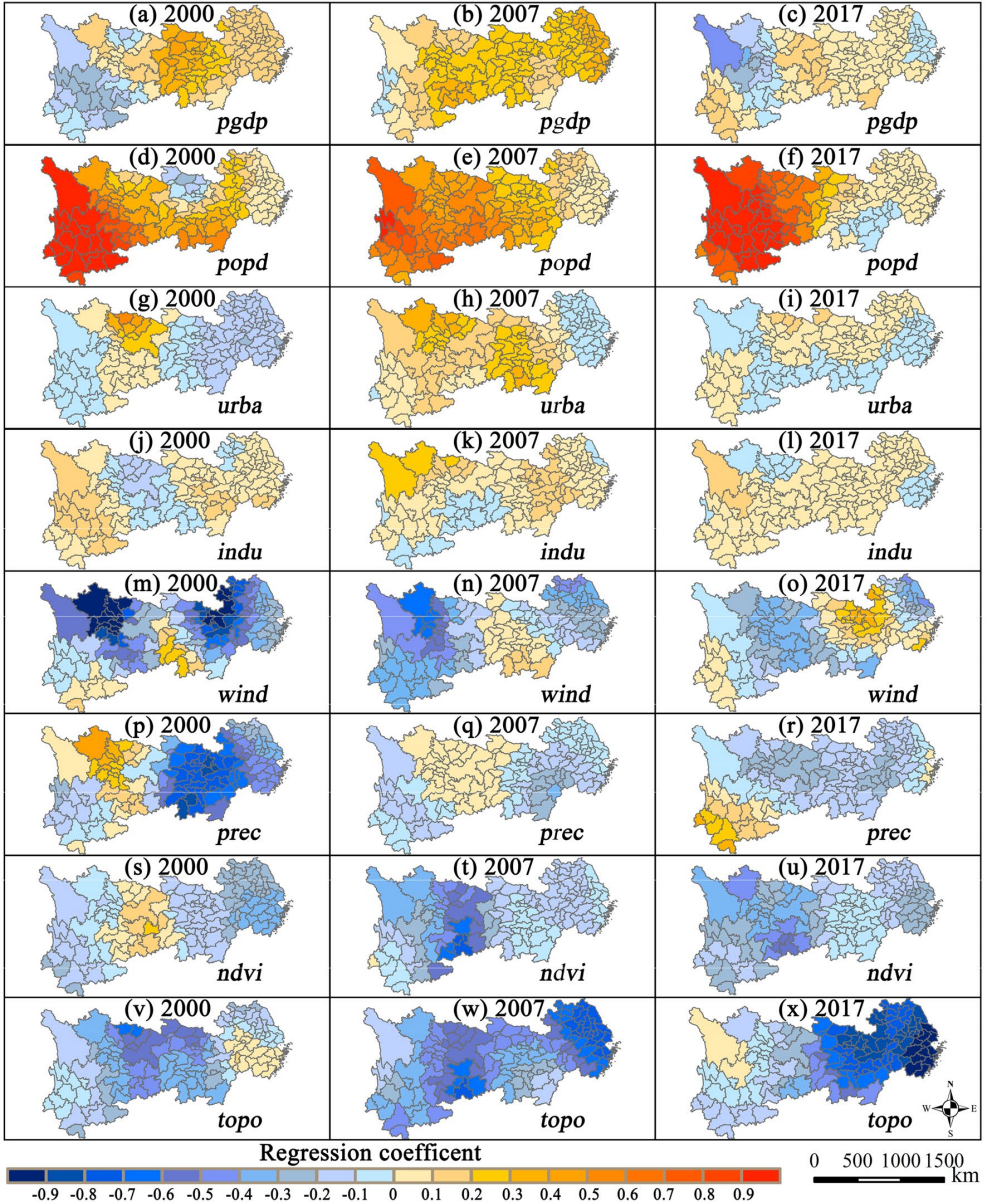
Spatial distribution of regression coefficients of the GWR model.

The coefficient of anthropogenic factors increased, signifying that extensive economic development and intensive human activities aggravated haze pollution. Existing studies have argued that economic growth strongly correlates with regional environmental pollution, but the relationship between GDP and PM_2.5_ is significantly different in various regions^19^. Per capita GDP had positive impacts on PM_2.5_ in the middle-upper reaches of the Yangtze River (Fig. 5a–c). This implied that PM_2.5_ in economically underdeveloped areas was more sensitive to economic development and that the economic growth of these regions came at the cost of the environment. Some cities in the YRD showed negative correlation effects, which indicated that the development planning in the above areas was relatively good. With technological progress, industrial upgrading and economic development, these areas were essentially coordinated with the surroundings.

The coefficient of population density showed approximately the same spatial distribution at the three time nodes (Fig. 5d–f), all of which increased from the coastal areas to the inland areas, and among them, the east–west difference was obvious in 2017. This may be because of the increase in urban traffic flow and production, with a higher population density contributing to an increase in the local PM_2.5_ level. The control of air pollution in the middle-upper reaches of the Yangtze Basin was still weak, thereby making the impact of population density more significant. Some studies have held that an increase in population density may have an agglomeration effect in promoting regional technical progress, thus facilitating a reduction in the local PM_2.5_ level^14^. The technical advances brought by the agglomeration effect for the population size in the YREB were not significant. This may be related to the migration of people to large cities in recent years and the disordered nature of population mobility.

The coefficient of the urbanization rate was positive at the three time nodes. The proportion of positive values in 2007 was relatively large, and the positive values in 2017 marginally declined (Fig. 5g–i). During 2000–2007, areas with low urbanization rates in the Yangtze Basin were experiencing rapid urbanization, and the urban infrastructure industry developed rapidly^9^. A large quantity of building dust entered the atmosphere, aggravating urban PM_2.5_ pollution. However, areas with a high urbanization rate, such as the YRD, tended to mature, and a stagnant infrastructure industry was conducive to the reduction in emissions of fine particles.

The industrial structure had a negative impact on PM_2.5_ in the middle-lower Yangtze and Cheng–Yu areas, aggravating local air pollution (Fig. 5j–l). This is consistent with the findings of existing studies confirming that industrial activities were the main drivers of PM_2.5_ in most areas^19^. The impact of the industrial structure in the Chengdu–Chongqing areas was strong, which may be because of heavy industries, such as the energy, chemical, and machinery industries, with relatively high direct energy consumption and pollutant emissions^25^. Optimization of the industrial structure significantly affects the local PM_2.5_ level. The coefficient had a weak impact on the YRD because the local industrial structure was dominated by the service industry and was relatively stable; thus, there was limited scope for further optimization.

The coefficients of wind in the inland areas were mainly positive and decreased from the central region to the west (Fig. 5m–o). The negative impact was dominant in the eastern areas, and as the distance to the coast decreased, the negative impact increased. This may be related to the impacts of topography and monsoons^27^. Coastal areas were mostly alluvial plains with flat terrains, and they were affected by the local circulation caused by monsoon and temperature differences. Clean air from the ocean had important dilution effects on pollutants, and thus, the coefficient was mainly negative. In the Sichuan Basin, the impact of closed topography restricted the diffusion of airflow, and the transport of wind caused pollutants in the region to interact with each other; thus, the coefficient was mainly positive. Similar findings were made in the Fenwei Plain, where basin topography exists^30^.

The impact of precipitation on regional PM_2.5_ presented a negative correlation at the three time nodes (Fig. 5p–r). A positive influence was mainly distributed in the Sichuan Basin and some areas of the Yunnan–Guizhou Plateau in western China, while the negative effect decreased from the coastal to inland areas. Concretely, the regions with a high regression coefficient were mainly located in the upper and middle reaches of the Yangtze Basin, while the eastern coastal areas with abundant rainfall had a small regression coefficient, indicating that abundant precipitation had a positive impact on PM_2.5_ in most cities, and this effect was more distinct in areas with relatively deficient precipitation.

The impact of the NDVI on PM_2.5_ had a negative correlation that was mainly distributed in the middle-lower Yangtze Plains (Fig. 5s–u). Research has shown that vegetation growth is correlated with climate and is affected by topography and human activities, resulting in a complex correlation between PM_2.5_ and vegetation^21^. The high-value areas were mainly in Yunnan and Hunan Provinces, while the low-value areas were concentrated in the middle-lower Yangtze Plain. In 2017, the NDVI showed a negative impact, indicating that vegetation’s inhibitory effect on PM_2.5_ was distinctly enhanced. This may be related to the rapid restoration and development of vegetation in the Yangtze Basin, whose forest coverage rate rose from 24% in 2000 to 39% in 2017^9^.

Topographic relief was negatively correlated with regional PM_2.5_ in the Yangtze Basin, which was conducive to the improvement in air quality (Fig. 5v–x). Specifically, the high-value regions were located in Sichuan, Chongqing, and Zhejiang. Lower values were concentrated in the middle-lower reaches of the Yangtze River. Local topography affects the diffusion and dilution of atmospheric pollutants in an area by influencing meteorological conditions^20^.

### Interactions between factors

Table 5 shows the results of the interactions between factors. The impact of the interactions between factors was greater than that for individuals, and the interaction effects included nonlinear enhancement and bifactor enhancement. When wind speed, precipitation, and vegetation interacted with PM_2.5_ in pairs, the nonlinear enhancement effect was generated at all three time nodes, and the explanatory power was notably varied in different periods. When the industrial structure interacted with the per capita GDP and urbanization level on PM_2.5_, a nonlinear enhancement effect was exerted at all three time nodes, and the explanatory power was continuously improved. Nonlinear enhancement means that the interactive impact of two factors is greater than the sum of the impacts when they act alone. The interactive types of *pgdp* ∩ *popd*, *pgdp* ∩ *urba*, *wind* ∩ *topo*, *prec* ∩ *topo*, and *ndvi* ∩ *topo* were dominated by nonlinear enhancement, although they varied at different times. The types of *popd* ∩ *urba* and *popd* ∩ *indu* exerted a bifactor enhancement effect, which was not as significant as that of the nonlinear enhancement.

**Table 5:** Results of interaction detecting.

Factors	2000	2007	2017	Factors	2000	2007	2017
*pgdp* ∩ *popd*	NE (0.682)	NE (0.745)	BE (0.709)	*wind* ∩ *prec*	NE (0.624)	NE (0.488)	NE (0.749)
*pgdp* ∩ *urba*	NE (0.281)	NE (0.420)	BE (0.421)	*wind* ∩ *ndvi*	NE (0.570)	NE (0.486)	NE (0.814)
*pgdp* ∩ *indu*	NE (0.370)	NE (0.225)	NE (0.472)	*wind* ∩ *topo*	NE (0.666)	NE (0.794)	BE (0.843)
*popd* ∩ *urba*	NE (0.654)	BE (0.757)	BE (0.748)	*prec* ∩ *ndvi*	NE (0.442)	NE (0.427)	NE (0.373)
*popd* ∩ *indu*	BE (0.657)	NE (0.747)	BE (0.762)	*prec* ∩ *topo*	NE (0.602)	BE (0.757)	NE (0.841)
*urba* ∩ *indu*	NE (0.397)	NE (0.432)	NE (0.516)	*ndvi* ∩ *topo*	NE (0.649)	NE (0.842)	BE (0.847)

*NE* nonlinear enhancement, *BE* bifactor enhancement.

## Conclusions and policy implications

### Conclusions

Haze pollution in Chinese cities has escalated to hazardous levels in recent years. This environmental problem has become a great challenge for public health and urban sustainable development. Our exploration of PM_2.5_ in the YREB provides useful results for haze prediction, which is an important step toward protecting people from health damage caused by poor air quality. The results indicate that the annual PM_2.5_ level of the YREB displayed an upward trend before 2007 and a fluctuating downward trend thereafter. PM_2.5_ had significant spatial heterogeneity and convergence characteristics. There were clear spatiotemporal differences in the impact of various factors on the PM_2.5_ pollution pattern. In the socioeconomic layer, population has the greatest impact, followed by the economy and industry, while urbanization was a relatively stable factor causing the rise in the PM_2.5_ level. Among the natural factors, topography and vegetation mainly exerted a negative impact on PM_2.5_, while the potency and direction of others changed with spatiotemporal changes.

### Policy implications

As the Yangtze River plays a vital role in China’s eco-environmental systems, stricter measures should be implemented to meet the goal of sustainable development. First, special attention should be given to natural factors when distributing industries and residences; for instance, topography and wind, which notably affect PM_2.5_, should be considered, while urban air ducts and green belt designs must be optimized. Second, region-targeted policies should be considered based on spatial differentiation. Downstream areas should play a leading role in promoting pollution prevention and control technologies, while upstream areas should actively drive ecological protection. More emphasis should be placed on transregional linkage governance when formulating mitigation measures. Third, heavily and highly polluted industrial sectors must be closed or upgraded, as they are major sources of particulates, while sectors with environmental protection, new energy, and a low-carbon economy and technology should be highly encouraged, such as building hi-tech eco-industrial parks. Last, it seems inevitable that air pollution will be aggravated because of burgeoning urbanization, and therefore, reducing the impact of anthropogenic activity on the atmosphere through environmental policy and education will reduce PM_2.5_ pollution.

### Limitations and recommendation

Our study mainly focuses on the exploration of the heterogeneity and determinants of PM_2.5_ at the macro-level. However, haze reduction ultimately needs to be implemented at micro-level enterprises, so investigations of micro-enterprises should be strengthened. In addition, PM_2.5_ is closely related to other air pollutants, which leads to compound air pollution with multiple pollutants. Therefore, it is necessary to explore the mechanism of compound pollution in the future to better formulate control measures.
